# Infant with protein C deficiency and stroke in the setting of iron deficiency anemia

**DOI:** 10.1002/ccr3.2271

**Published:** 2019-07-23

**Authors:** Tahseen Jalal Karim, Dustin J. Paul, Regina M. Troxell, Rajan Patel, Ian J. Butler

**Affiliations:** ^1^ McGovern Medical School Houston Texas

**Keywords:** iron deficiency, pediatrics, protein C deficiency, stroke

## Abstract

We report an 18‐month‐old infant with ischemic stroke, neurocognitive impairment, and psychomotor retardation in the setting of severe iron deficiency anemia. Although an uncommon outcome in anemic children, stroke is important to consider as a cause for developmental delay in children with iron deficiency anemia.

## 1 INTRODUCTION

An estimated 114 million children aged 0‐5 years old worldwide have iron deficiency anemia making it the most common nutritional disorder.^1^ Most children with iron deficiency anemia are asymptomatic, but pallor, fatigue, and jaundice are common presentations. Geophagia and pica (the craving and consumption of soil, dirt, or clay) are atypical phenomena that have been reported. Chronic iron deficiency anemia has been associated with impaired behavioral, cognitive, and motor development.^2^, ^3^

Ischemic stroke is a rare manifestation of iron deficiency anemia. Its pathogenesis in anemic children is not well understood. Most childhood strokes present with focal neurological deficits while some may produce developmental delay.^3^ However, there is no definitive link between iron deficiency anemia and developmental delay.

We report an 18‐month‐old infant with ischemic stroke, neurocognitive impairment, and psychomotor retardation in the setting of severe iron deficiency anemia. Laboratory values support the diagnosis of iron deficiency anemia, while imaging and clinical examination confirm the presence of infarct within the brain. Although it is an uncommon outcome in anemic children, it is important to consider stroke as a cause for developmental delay in children with iron deficiency anemia.

## 2 PATIENT DESCRIPTION

An 18‐month‐old male presented to the emergency department with five days of high‐grade fever (*T*_max_ 102.3°F), cough, congestion, rhinorrhea, and dyspnea. The patient's mother also noted swelling and decreased movements of the right upper extremity the day before admission. He had no contributory past medical history. The mother had an uncomplicated pregnancy and good prenatal care. Birth history was uneventful with cesarean section at 41‐weeks gestation.

The infant had poor follow‐up with his pediatrician and had missed several vaccinations. Since birth, his diet was poor consisting of cow's milk and sparse solid foods. His motor and language development were delayed. He began crawling at 8 months, pulled to stand at 15 months, and had never walked at the time of admission. The only words he spoke were “mama” and “papa.” Family history was significant for childhood strokes in his maternal grandmother and maternal grandaunt.

Upon admission, the patient was mildly tachycardic at 148 beats per minute, but all other vital signs were within normal limits. On physical examination, he appeared somnolent, pale, and malnourished (weight, 10.52 kg‐11th percentile and length, 80 cm‐15th percentile). Cardiovascular examination was normal. On pulmonary examination, subcostal retractions were present, and rales were heard in the base of the left lower lobe. Abdominal examination was normal.

On neurological examination, cranial nerves II‐XII and gross sensation in all extremities were intact. Deep tendon reflexes were 2+ in all extremities. Plantar reflexes were upgoing bilaterally. The left upper and bilateral lower extremities showed full range of motion. There was no active movement of the right upper extremity even after painful stimulation of the right hand. The right upper extremity was hypotonic, but tone was normal and symmetrical in all other extremities. There were no abnormal movements or tremor.

A comprehensive metabolic panel showed a serum chloride of 113 mmol/L, calcium of 7.8 mg/dL, phosphorus of 3.0 mg/dL, total protein of 4.8 g/dL, albumin of 1.9 g/dL, and a total bilirubin of 0.4 mg/dL. Lactate dehydrogenase was 340 IU/L.

A complete blood count showed platelet count of 177 × 10^3^/µL, red blood cell count of 2.2 × 10^6^ cells/µL, hemoglobin of 2.4 g/dL, hematocrit of 10.0%, mean cellular volume of 46.1 fL, mean corpuscular hemoglobin concentration of 23.8 g/dL, and red blood cell distribution width of 26.3%. An iron panel showed serum iron of 8 µg/dL, ferritin of 8 ng/dL, iron saturation of 2%, and total iron binding capacity of 351 µg/dL.

The patient was immediately transfused three units of packed red blood cells and given ferrous sulfate. With concern for pneumonia, a chest X‐ray was performed revealing left lower lobe infiltrates. Broad‐spectrum treatment was started with ampicillin. Blood and urine cultures were negative.

**Figure 1:**
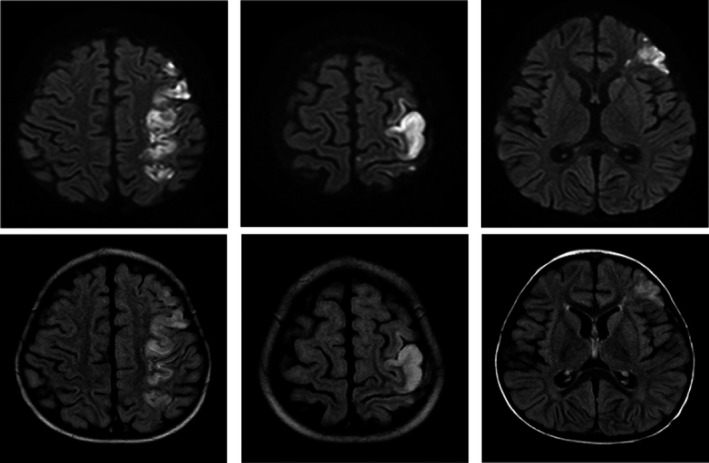
Magnetic resonance imaging of abnormal restricted diffusion/infarct within the left middle cerebral artery and watershed distribution (day 4 of hospitalization). DWI (top) and T2 FLAIR (bottom)

**Figure 2:**
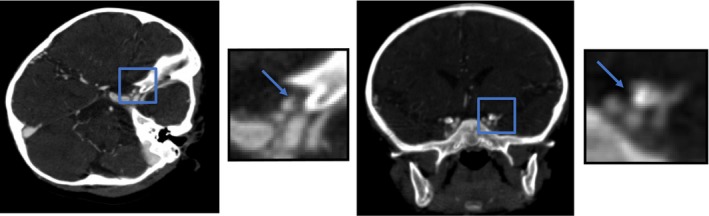
Computer tomography angiogram of small crescent‐shaped luminal irregularity (zoomed in, as depicted by arrow) within the ophthalmic segment of the left internal carotid artery with possible small filling defect/thrombus or focal intimal flap

**Figure 3:**
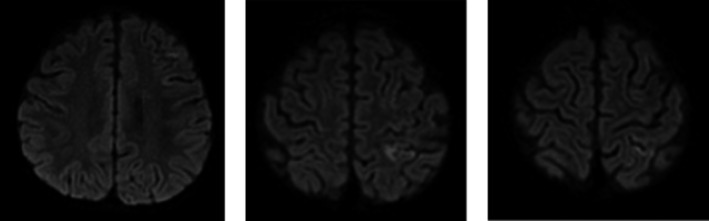
Magnetic resonance imaging of interval near complete resolution of previously seen abnormal restricted diffusion/infarct within the left middle cerebral artery and watershed distribution (day 12 of hospitalization)

Suspicion for stroke led to magnetic resonance imaging of the brain which exhibited restricted diffusion in the left middle cerebral artery (ie, left cortical) and watershed distribution (Figure 1). A four‐extremity venous Doppler did not identify any deep vein thrombosis or other potential source of embolus. Magnetic resonance venography of the brain showed patent venous sinuses throughout. A computed tomography angiogram of the head and neck demonstrated a luminal irregularity and possible filling defect in the cavernous segments of the left internal carotid artery (Figure 2). Magnetic resonance imaging on day 12 of hospitalization showed resolution or changes of the ischemia (Figure 3).

Thrombophilia studies showed a prothrombin time of 14.7 seconds, international normalized ratio of 1.13 partial thromboplastin time of 16.5 seconds, protein C of 41%, protein S of 74%, homocysteine of 3.4 µmol/L, and negative prothrombin and factor V mutations. The patient developed a rash after acetylsalicylic acid administration so he was given clopidogrel instead.

Right bundle branch block and signs of right ventricular hypertrophy evident on electrocardiogram necessitated further cardiology workup. A transthoracic echocardiogram found no evidence of right heart strain, systolic dysfunction, or intracardiac vegetations or thrombi. A small patent foramen ovale with left to right shunting was found.

His pneumonia eventually resolved, and on day 14 of hospitalization, he was discharged on iron supplementation and clopidogrel. Following discharge home, he began to drink less cow's milk and eat regular foods. Over time, he regained strength and tone of his right arm. However, his development continued to be delayed. At 24 months, he still did not say more than a few words and was only able to take a few steps. Follow‐up laboratory tests 7 months after his hospitalization were normal except for persistently low protein C of 47%. Whole exome sequencing is still pending.

## 3 DISCUSSION

Our patient presented with severe iron deficiency anemia most likely secondary to excessive cow's milk consumption, a common cause of iron deficiency anemia. Low hemoglobin, mean cellular volume, serum iron, and ferritin and high a red blood cell distribution width and total iron‐binding capacity on laboratory studies support the diagnosis severe microcytic anemia due to chronic iron deficiency. The most frequently reported symptoms in anemic children are pallor and fatigue.^2^ Stroke is a relatively uncommon manifestation of iron deficiency anemia.^3^, ^4^ Yet, previous case‐control studies have suggested that children who developed stroke were about 10 times more likely to have iron deficiency anemia than healthy children who did not develop stroke.^4^ The pathophysiology of ischemia due to iron deficiency is not well understood. Several hypotheses have been proposed, including low serum iron levels leading to thrombocytosis, hypercoagulable states, and anemic hypoxia.^3^

Iron has been shown to regulate megakaryocyte and platelet production, possibly through direct or indirect inhibition of thrombopoietin and erythropoietin. Iron deficiency may lead to the loss of inhibition of thrombopoiesis resulting in reactive thrombocytosis.^5^, ^6^ Low serum iron levels have also been shown to increase the expression of megakaryopoietic markers and enhance proplatelet formation.^6^ However, the association between high platelet levels and thrombosis is not very clear. There have been several cases in which anemic children with thrombosis did not have thrombocytosis.^3^, ^7^, ^8^ Our patient had normal platelet levels that remained stable throughout the hospital admission. Untreated iron deficiency anemia has also been reported to result in thrombocytopenia.^3^

Thrombus formation requires platelet activation, platelet aggregation, and fibrin deposition, processes which are amplified in a hypercoagulable state.^7^ Iron deficiency induces a microcytosis in which RBCs are less deformable leading to increased blood viscosity. Such conditions may cause turbulent blood flow and endothelial damage facilitating abnormal platelet aggregation.^3^, ^8^ According to a study by Tekin et al, iron deficiency may also reduce antioxidant protection which can increase platelet activation and aggregation.^9^

Pediatric patients with iron deficiency anemia have presented with cerebral venous thrombosis.^3^, ^10^ In our patient, neither four‐extremity venous Doppler nor magnetic resonance venography showed evidence of venous thrombosis. However, computed tomography angiogram revealed an equivocal region of occlusion within the left internal carotid artery. Intraluminal thrombi of the internal carotid artery causing stroke and multiple transient ischemic attacks in patients with iron deficiency anemia have been previously reported.^7^, ^10^ Iron deficiency anemia can further worsen hypoxia in regions of decreased cerebral perfusion.^3^, ^10^ Typically affected regions include watershed areas (ie, zones between two nonanastomosing arterial systems) distal to a thrombosed internal carotid artery, similar to the location of infarct identified in our patient. Increases in metabolic demand during infection can also worsen hypoxia.^3^, ^10^ Our patient presented with a pneumonia that may have elicited systemic stress producing a state of inadequate oxygen‐carrying capacity in an already vulnerable watershed area.

At initial presentation, our patient had a previously undiagnosed and asymptomatic protein C deficiency. Protein C deficiency is an acquired or hereditary (ie, congenital) deficiency of the anticoagulant protein C and is associated with increased risk of deep vein thrombosis, cerebral venous thrombosis, pulmonary embolism, and/or arterial stroke.^11^, ^12^, ^13^ The disease can be transient most commonly associated with disseminated intravascular coagulation, sepsis, malignancy (eg, AML), hepatic dysfunction, respiratory distress syndrome, and heart failure, none of which our patient had.^11^, ^13^ Children with transient protein C deficiency have presented with venous and intracardiac thromboses.^11^ Our patient had no family history of diagnosed protein C deficiency or other hypercoagulable disease. However, childhood strokes of unknown etiology on the maternal side of the family were reported. On follow‐up laboratory testing, our patient continued to show a low protein C level, implicating an acquired or congenital deficiency. Children with protein C deficiency may also present with developmental delay.^13^, ^14^ To the best of our knowledge, there is no literature describing the relationship between iron deficiency anemia and protein C deficiency nor the occurrence of thrombosis in patients who have both deficiencies concurrently.

Iron deficiency anemia has been associated with severe delays in behavioral, cognitive, and motor development.^2^, ^3^ Silent cerebral infarcts are defined as areas of ischemia identified on magnetic resonance imaging of the brain that do not produce obvious neurologic deficits. Silent cerebral infarct is the most common manifestation of neurologic disease in children with sickle cell disease.^15^, ^16^ However, Dowling et al found that hospitalized children with sickle cell disease and silent cerebral infarct (identified by magnetic resonance imaging) had lower cognitive test scores and impaired global intellectual function compared with children with normal magnetic resonance imaging.^15^, ^16^ Children without sickle cell disease but severe anemia showed silent cerebral infarcts; however, none of those children had iron deficiency anemia. Multiple and/or repetitive silent strokes may explain neurocognitive and psychomotor delay in children with nutritional deficiencies, including iron deficiency. Some silent cerebral infarcts have been shown to resolve with time and treatment as did the ischemia observed in our patient.^16^

## 4 CONCLUSION

Hypercoagulability and platelet aggregation secondary to iron deficiency anemia and/or protein C deficiency likely resulted in thrombosis and ischemic stroke in our patient. Further studies are necessary to determine the association between low levels of iron and protein C to explain the pathophysiology of thrombosis in the setting of iron deficiency anemia and protein C deficiency. Furthermore, determining the true incidence of stroke or silent cerebral infarct in children with iron deficiency anemia may help identify a causal relationship between iron deficiency anemia and developmental delay. Nonetheless, our patient illustrates the importance to consider stroke in children with iron deficiency anemia that show signs of neurocognitive and psychomotor impairment.

## CONFLICT OF INTEREST

None declared.

## AUTHOR CONTRIBUTION

TJK: wrote and edited manuscript. DJP: acted as physician of patient and contributed to and edited manuscript. RMT: contributed to and edited manuscript. RP: reviewed and provided imaging. IJB: acted as lead physician and contributed to and edited manuscript.
